# Seroprevalence and risk factors for SARS-CoV-2 infection in middle-sized cities of Burkina Faso: A descriptive cross-sectional study

**DOI:** 10.1371/journal.pone.0305850

**Published:** 2024-08-07

**Authors:** Adama Sana, Elodie Djemaï, Philippe De Vreyer, Thomas Thivillon, Hermann Badolo, Abdramane Berthé, Dramane Kania

**Affiliations:** 1 Département biomédical et santé publique, Institut de Recherche en Sciences de la Santé (IRSS), Centre National de la Recherche Scientifique et Technologique (CNRST), Ouagadougou, Burkina Faso; 2 Université Paris-Dauphine, Université PSL, LEDa, CNRS, IRD, DIAL, Paris, France; 3 Université de Bordeaux, UMR CNRS 6060 Bordeaux Sciences Economiques, Pessac, France; 4 Observatoire National de la Santé de la population, Institut National de Santé Publique, Ouagadougou, Burkina Faso; 5 Université de Dédougou, Dédougou, Burkina Faso; 6 Centre Muraz, Institut National de Santé Publique, Bobo-Dioulasso, Burkina Faso; CEA, FRANCE

## Abstract

**Background:**

Since March 2020, COVID-19 has evolved from a localized outbreak to a global pandemic. We assessed the seroprevalence of COVID-19 in three towns in the Centre Sud region of Burkina Faso.

**Methods:**

A population-based cross-sectional survey was conducted in three middle-sized cities in Burkina Faso’s Centre Sud region, from June to July 2021. Subjects aged 16 or over at the time of the survey were considered for this seroprevalence study. The Biosynex COVID-19 BSS rapid test was used to detect immunoglobulin G (IgG) and immunoglobulin M (IgM) against SARS-CoV-2. A standardized questionnaire was also administered to collect additional information.

**Results:**

A total of 2449 eligible participants (age ≥ 16 years) were identified. Serological tests for COVID-19 were performed in 2155 individuals, of which 2143 valid tests were retained and analyzed. Out of the entire sample, 246 positive tests were observed, corresponding to a prevalence of 11.48%. Prevalence was 9.35% (58 cases) in Kombissiri, 12.86% (80 cases) in Manga and 11.99% (108 cases) in Pô. By gender, 13.37% of women (164 cases) tested positive, and 8.95% of men (82 cases). Women accounted for 66.67% of all positive test subjects. The results from the multivariate analysis show a significantly higher seroprevalence in women (p = 0.007), people over 55 years old (p = 0.004), overweight people (p = 0.026) and those with drinking water sources at home (p = 0.013).

**Conclusions:**

The results of this study show that the COVID-19 virus also circulates in the population of middle-sized cities in Burkina Faso, far more than officially reported by the information service of the government of Burkina Faso, given the lack of systematic testing in the general population in the country. The study also highlighted the greater vulnerability of women, older and overweight individuals to the epidemic. The preventive measures put in place to fight the pandemic must take these different factors into account.

## Introduction

Coronavirus disease 2019 (COVID-19) is an infectious disease whose initial cases were first reported in Wuhan, Hubei Province, China, in December 2019 [1]. Rapidly evolving from a localized outbreak, it transformed into a global pandemic [2]. In Africa, the first case of COVID-19 was reported on February 14, 2020 in Egypt [3]. Sub-Saharan Africa (SSA) experienced its first case on February 27, in Nigeria [4, 5]. Two months later the disease had spread to every country in Africa.

Data from the countries first affected by the pandemic indicate that around 40% of infected people have a mild form of the disease, often asymptomatic [6]. Despite the absence or presence of mild signs and symptoms, these people are contagious and therefore capable of transmitting the disease to others [6].

Early in the pandemic, scientists predictions suggested that the African continent would be the most negatively affected [7, 8]. Indeed, fragility of health care systems, limited access to hygiene and sanitation facilities, and lack of early treatment options during the initial stages of the pandemic raised concerns about a potentially rapid increase in infections in sub-Saharan Africa. Against all expectations, African countries have not had the high rates of COVID-19 cases, hospitalizations and deaths that had been predicted [9]. Indeed, approximately 3 years after the 1st case was confirmed in Africa, the WHO reported, in its bulletin of February 24, 2023, 10.8 million COVID-19 cases on the continent, with 228,738 deaths. Africa accounted for 1.3% of cases reported globally (757.2 million) and 1.2% of deaths (6.8 million) [10]. The Republic of South Africa was the most affected African country. For some authors, the low burden of COVID-19 in Africa can be explained by the younger age of its population (low mortality, more asymptomatic cases because of the tolerance of the disease by young people) [11], the high prevalence of parasitics diseases (reduces the virulence of the SARS-CoV-2 by restricting the hyper-inflammation associated with the viral infection, resulting in high numbers of asymptomatic cases) [11–13], the history of tuberculosis vaccination, traditional medicine complementing poor healthcare systems, population movements (low air traffic exchanges compared with other continents), climate/temperatures, etc. Other authors argue that the low burden of COVID-19 could be explained by the absence of detection (limited testing capacity) and reporting systems in most African countries [4, 14].

In several African countries, such as Burkina Faso, diagnostic tests were carried out on travellers, symptomatic people and contacts of people who tested positive. Because of rumors about hospitalization conditions for COVID-19 cases, some people avoided visiting health facilities or calling the toll-free number if they had symptoms of the disease [15]. Some then turned to traditional medicine, parapharmaceutical solutions (Artemisia, Apivirine, etc.) and foods (garlic, ginger, turmeric, lemon, honey, etc.) for prevention and treatment of COVID-19 [15–17].

Burkina Faso is a low-income Sahelian country, ranked 184th out of 191 countries in the United Nations Development Programme’s (UNDP) Human Development Index 2021–2022 report [18]. Since 2015, the country has been the target of terrorist attacks that have led to population displacements and have been at the root of several successive coups. Over 40% of the population lives below the poverty line. Some studies reported that social and economic factors such as gender, education, income, place of residence (big city, middle-sized town or village), ethnicity, underlying medical conditions and living in densely populated areas, correlate with the risk of being affected by COVID-19 in terms of both incidence and mortality [19].

Burkina Faso confirmed its first case of SARS-CoV-2 infection on March 15, 2020 [5]. By July 3, 2021, the end of the investigation on which this article is based, the total number of cumulative cases in Burkina Faso was 13,453 [20]. By July 3, 2022, 21,134 cases had been officially confirmed, and 387 patients had died because of the disease [20].

On March 13, 2020, the Government started disseminating public-awareness messages on COVID-19. Given the expansion of the pandemic, the Government announced public health containment and mitigation measures including the closure of education facilities, the closure of air and land borders, the introduction of a curfew between 7pm and 5am, a ban on gatherings of more than 50 people, the closure of markets, social distancing guidelines and the mandatory use of masks [15].

Vaccinations against COVID-19 started in Burkina Faso on June 2, 2021 [21]. The country received 115,000 doses of COVID-19 vaccine on May 30, courtesy of COVAX [22]. Priority targets were healthcare workers, people with co-morbidities and candidates for the pilgrimage to Mecca. On December 20, an intensive vaccination campaign against COVID-19 was launched with the aim of reaching at least 10% of the population aged 18 and over by the end of December 2021 [21]. However, this campaign only involved four of the country’s thirteen regions, as they were identified as the epicenter of the disease: Centre, Centre-Ouest, Hauts-Bassins and Sud-Ouest regions [21]. Vaccination against COVID-19 began in the Centre-Sud region with the publication on January 7, 2022 of an information note containing the list of COVID-19 vaccination sites by the Ministry of Health and Public Hygiene (Page Facebook https://web.facebook.com/MshpBurkina/posts/liste-des-sites-de-vaccination-contre-la-covid-19-de-la-r%C3%A9gion-du-centre-sud/2998057370509133/?_rdc=1&_rdr). Hence the sample in our study was not yet eligible for vaccination at the time of the survey. The majority of officially confirmed cases have occurred in Ouagadougou capital-city, situated in Burkina Faso Centre region, and Bobo-Dioulasso, the country’s second-largest city located in the Hauts-Bassins Region [15, 23]. The Centre Region alone accounts for 84.2% of the cumulative number of cases observed throughout the country, followed by the Hauts-Bassins region with 8.9% of cases [15]. As a result, these two cities were targeted and benefited the most from response measures.

To get a more objective idea of the extent of the disease, a few studies on SARS-CoV-2, including seroprevalence studies, have been carried out in Ouagadougou and Bobo-Dioulasso [24–26]. To the best of our knowledge, population-based seroprevalence studies on SARS-CoV-2 infection in middle-sized towns remain rare, if not non-existent.

This study aims to assess the epidemic situation in middle-sized towns in the Centre-Sud region of Burkina Faso and to identify the risk factors for COVID-19 infection. Previous evidence on these middle-sized towns remain scarce at the time of our study, partially due to socio-cultural constraints, socio-demographic characteristics (higher household size), limited financial resources, poorly-equipped healthcare facilities and medical staff unfamiliar with the management of this new pathology [15].

## Materials and methods

### Study site

**Fig 1:**
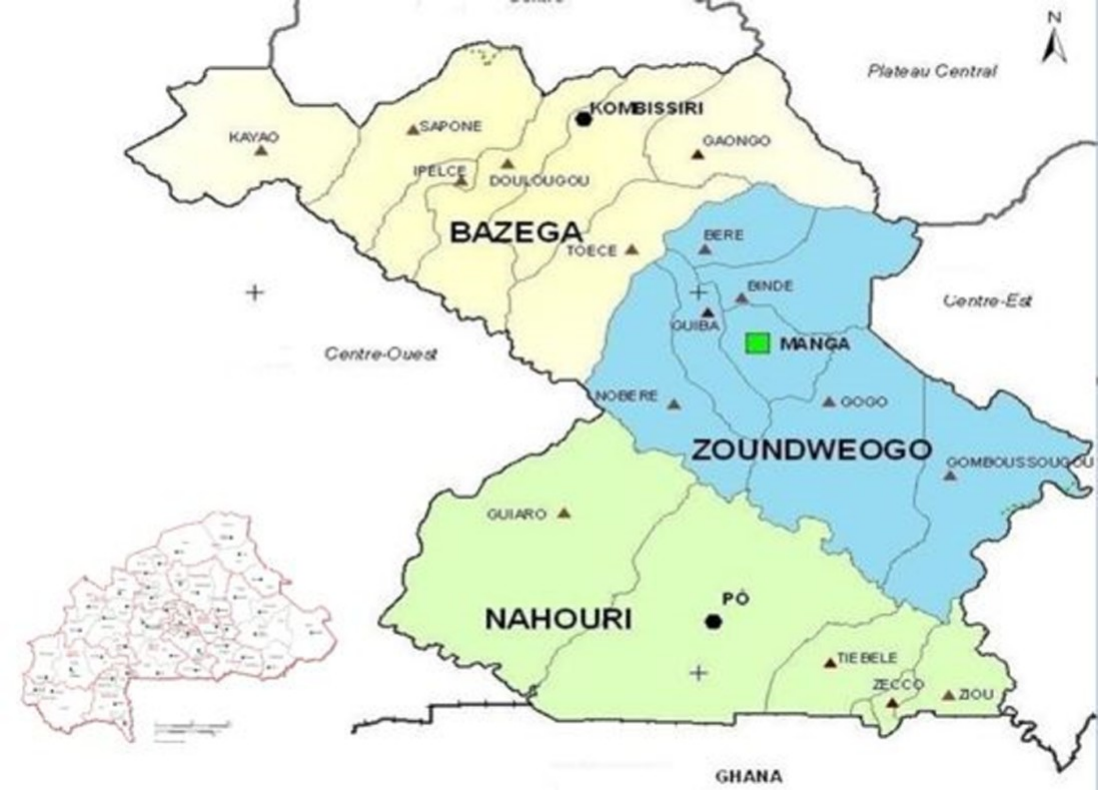
Map of the study site.

Burkina Faso is divided into 13 administrative regions (since 2001), which in turn are administratively divided into 45 provinces. The study took place in the Centre-Sud region of Burkina, located in the south of Ouagadougou on the way to Ghana. The Centre-Sud region is composed of three provinces: Bazèga, Nahouri and Zoundwéogo (as shown in the map in Fig 1). To each of the 45 provinces of Burkina Faso is associated a “chef-lieu” (i.e. a main town, administrative center). Kombissiri is the “chef-lieu” of Bazèga, and Pô and Manga the “chef-lieu” of Nahouri and Zoundwéogo respectively. According to the last census data collected in 2019 [27], the size of the population is 28,617 in Kombissiri (national ranking = 29), 28,615 in Manga (rank = 30) and 28,079 in Pô (rank = 31). These three cities can therefore be considered middle-sized, compared with Ouagadougou (2,415,266) and Bobo-Dioulasso (904,920).

### Study participants

This study is a descriptive cross-sectional investigation based on survey data collected from June 7 to July 3, 2021. It relies on a preliminary study on domestic cooking fuel choices and exposure to air pollution started in 2019, before the epidemic of COVID-19. As the mentioned study focused on the adoption of clean cookstoves (stoves using gas or electricity, as opposed to other fuels like charcoal or wood) by poor households, the first phase was carried out on a random sample of households with no access to gas or electricity for cooking. The choice of study site stems from the fact that access to clean cookstoves was limited in these cities at that time [28].

The sample of households comes from a list of households that had been surveyed between November 2019 and March 2020. Households were initially randomly selected using a spatial sampling strategy, with GPS points drawn in the three study locations. Enumerators followed a predetermined random walk to select one household per GPS point. The sample size was initially determined to detect an 8.5 percentage points increase in the proportion of households cooking with a clean cooking appliance (gas stove) with a power of 0.8 and statistical significance at the 1% level, taking into account an anticipated attrition rate of 3%. The corresponding sample size was 820 households.

The current analysis relies on 813 households. 739 had already been surveyed in the first survey prior to COVID-19, and 74 are replacement households. 229 located in Kombissiri, 233 in Manga and 351 in Pô.

### Inclusion and exclusion criteria

There are two exclusion criteria in the sample used in the paper.

First, eligible households were those without access to gas or electricity for cooking. In addition, those declaring dolo brewing (that involves the use of very large quantities of wood as fuel and generates very high level of fine particle emissions) as one of their income-generating activities, or reporting no cooking at home were excluded from the study. These restrictions are limited because most households in Burkina Faso rely on solid fuels for cooking. Data from the living standard measurement surveys collected in 2014 [29] show that 83% of the households rely on wood as primary cooking fuel at the national level, and only 13.4% have access to a cleaner cooking solution such as LPG or electricity.

Second, the individual sample is restricted to household’s members aged 16 and older because the serological tests were only conducted among adults. We exclude people aged under 16 because the acceptance was known to be more difficult to obtain from the parents of young children. People were included regardless of symptoms or risk groups.

The analysis sample consists of 813 households with at least one member tested for COVID-19 during our survey and with a valid test.

### Ethics statement

The project obtained approval from the Burkina Faso Health Research Ethics Committee on July 1, 2020 (Ethical clearance registration number: N°2020-7-132), and from the International Review Board of the Paris School of Economics on March 16, 2021 (Ethical clearance registration number: 2019–011).

An information form outlining the terms and conditions of the research was read and explained to the members of the sampled households (either in French, Kassena or Moore). Only after ensuring that the information contained in the information form was fully understood, was the participant’s consent requested by the data collector. To participate in the study, free and informed consent signed by the head of household or his/her representative was required. This consent was in both electronic (on tablet) and written (on paper) format. A signed and dated copy was given to the household before the start of the interviews. In addition, free and informed verbal consent was requested from each respondent. For minors, the consent of a parent or legal guardian was mandatory.

Participation was voluntary and each participant was notified of his or her right to terminate it at any time, without having to provide any reason or suffer any prejudice.

### Data collection

As part of this study we administered questionnaires and took capillary blood samples for COVID-19 rapid tests. The data collection started on June 7, 2021 and ended on July 2, 2021. Household members were interviewed using a standardized electronic questionnaire. The SurveyCTO software was used for questionnaire programming and data recording on tablets, including serological test results. Data collection covered sociodemographic characteristics, self-reported health, weight, height, and COVID-19 serological testing. For COVID-19 serological testing, a capillary blood sample was taken from study participants. The rapid test used was the Biosynex COVID-19 BSS, an immunochromatographic test that detects anti-SARS-CoV-2 IgG and IgM antibodies. The Biosynex COVID-19 BSS has been shown to have 91.8% (100%) sensitivity and 99.2% (99.5%) specificity for the IgM (IgG) by the reference center of Institut Pasteur when using RT-PCR tests as the reference method for clinical diagnosis in 446 blood samples for the IgG and 456 blood samples for the IgM (the report is available upon request).

### Measurement

The analysis was primarily descriptive, with an analytical component considering sociodemographic variables that could potentially influence COVID-19 serological status.

The measure of seroprevalence relies on the results from the serological tests. The prevalence rate is defined as the proportion of tested people who have SARS-CoV-2 antibody in their blood at the time of the data collection (either IgM or IgG). We use the terms prevalence, seroprevalence, or SARS-CoV-2 antibody prevalence with no distinction.

### Statistical methods

Data cleaning and analysis were performed using STATA (version 16.0). Categorical and continuous variables were described as frequency (%) or mean (M) ± standard deviation (SD). To assess the independent association of significant factors with a positive test, odds ratios (ORs) and their respective 95% confidence intervals (95% CI) were determined using bivariate and multivariate logistic regression models. To determine the ORs using logistic regressions, the dependent variable is a binary variable equal to one if the test is positive, and 0 if the test is negative.

To study the factors associated with a positive serological test, logistic regression analysis was conducted to investigate the relationship between being found positive to the serological test, and standard sociodemographic characteristics (sex, age, living conditions) and COVID-19 known risk factors (body mass index). In the multivariate analysis, we included all variables that had a significant association with SARS-CoV-2 infection (those with a critical probability below 0.10), except for gestational status, which was not included to analyze the complete sample of men and women.

## Results

### Sociodemographic characteristics of the study population

A total of 823 households were covered in the survey, encompassing 3,834 individuals, with 1,161 in Kombissiri, 937 in Manga, and 1,736 in Pô. Approximately 61% of households had more than five members. On average, 2.6 people were tested per household (median 2, min 1, max 12).

Our analysis sample comprises 2,143 household members aged 16 and over for whom we have a valid test. 1,227 are women and 916 are men. Among the women, 38 declared they were pregnant at the time of testing.

The mean age was 38.56 years (SD 17.46; min 16, max 111), with 38.76 years (SD 17.25; min 16, max 111) for women and 38.28 years (SD 17.74; min 16, max 92) for men. Approximately 56.40% of participants had never attended school. 44.33% lived in households with 1 to 5 members, and 7.41% had access to piped water (either inside their dwelling or from a neighbor). 66.04% had a normal body mass index (BMI), 15.83% were overweight, and 6.03% were obese (BMI ≥ 30). The descriptive statistics of the main variables are shown in Table 1.

**Table 1:** Descriptive statistics of the main sociodemographic variables.

Characteristic	Frequency	Proportion (%)
**Town** (n = 2,143)		
Kombissiri	620	28.93
Manga	622	29.03
Pô	901	42.04
**Sex** (n = 2,143)		
Female	1227	57.26
Male	916	42.74
**Age** in years (n = 2,143)		
16–24	599	27.95
25–54	1123	52.40
≥55	421	19.65
**Body Mass Index (BMI)** (n = 2,141)		
Underweight	259	12.10
Normal	1414	66.04
Overweight	339	15.83
Obese	129	6.03
**Education attainment** (n = 2,108)		
No formal education	1189	56.40
Any formal education	919	43.60
Primary	361	17.13
Lower Secondary	336	15.94
Upper Secondary and above	222	10.53
**Household size** (members) (n = 2,143)		
1–5	950	44.33
>5	1193	55.67
**Access to water**^**a**^ (n = 2,131)		
Yes	158	7.41
No	1973	92.59

Household members aged 16 or over with a valid rapid diagnostic test were included.

^a^Access to water is an indicator taking the value 1 if the household has access to clean water in its house, garden or yard, or from a neighbor.

### COVID-19 seroprevalence

Serological tests for COVID-19 were carried out on 2,155 of the 2,449 eligible individuals (aged 16 and over). The difference between the size of the eligible population and the size of the tested population is mainly due to absence at the time of data collection. The household members are all listed in the census but some of them were absent at the time of the survey. Of the eligible persons present, only two refused to give a blood sample for the COVID-19 test.

Among all tests made, only 12 tests were considered invalid due to the absence of the control line (C) on the cassette. Eventually, 2,143 valid tests were retained.

Across the entire sample of 2,143 valid tests, 246 positive tests were observed, resulting in a prevalence of 11.48%.

Prevalence was 9.35% (58/620) in Kombissiri, 12.86% (80/622) in Manga, and 11.99% (108/901) in Pô.

Among the 246 positive cases, 164 were women (prevalence rate of 13.37%, 164/1227) and 82 were men (prevalence rate of 8.95%, 82/916). Consequently, women constituted 66.67% of all subjects tested positive (164/246). Among pregnant women, the seroprevalence was 26.32% (10 cases out of 38).

The mean age of those tested positive was 41.22 years (SD 18.70; min 16, max 92). Seroprevalence was 10.51% (181 cases over 1,722) in individuals under 55 and 15.44% in those aged 55 and over (65 cases over 421).

### SARS-CoV-2 antibody prevalence and sociodemographic indicators

#### Univariate analysis

We compared seropositivity according to city of residence, household size, sex, age group, education level, and access to water (Table 2). Univariate analysis from logistic regression did not show a significant difference between the three cities (p = 0.050 and 0.107), household size (p = 0.779), or education level (p = 0.790, a variable indicating whether one was educated or not, observed in 2,108 respondents). However, a statistically significant difference was observed for gender (p = 0.002), high BMI (p = 0.005, variable indicating whether BMI is equal or greater than 25), age groups (p = 0.005), and gestational status among the 1,227 women (p = 0.021). In addition, there was a significant difference depending on whether the person was a member of a household with access to water access or not, with a p-value of 0.006.

**Table 2:** Factors associated for SARS-CoV-2 infection in the towns of Kombissiri, Manga, and Pô, in the Centre Sud region of Burkina Faso: Univariate analysis.

Variables	% Positive	Univariate OR	95% CI	P-value
**City (n = 2,143)**				
Kombissiri	9.35	1	Reference	Reference
Manga	12.86	1.43	1.00–2.05	0.050*
Pô	11.99	1.32	0.94–1.85	0.107
**Household size (n = 2,143)**				
1–5	11.26	1	Reference	Reference
>5	11.65	1.04	0.79–1.36	0.779
**Education (n = 2,108)**				
No formal education	11.32	1.04	0.79–1.36	0.790
Any formal education	11.69	1	Reference	Reference
**Sex (n = 2,143)**				
Female	13.37	1.57	1.19–2.08	0.002***
Male	8.95	1	Reference	Reference
**BMI group (n = 2,141)**				
BMI <25	10.46	1	Reference	Reference
BMI larger than 25	15.17	1.53	1.14–2.06	0.005***
**Age (n = 2,143)**				
16–54	10.51	1	Reference	Reference
55+	15.44	1.55	1.14–2.11	0.005***
**Water access (n = 2,131)**				
Yes	18.35	1.82	1.19–2.79	0.006***
No	11.00	1	Reference	Reference
**Currently pregnant (n = 1,227)**				
Yes	26.32	2.40	1.14–5.04	0.021**
No	12.95	1	Reference	Reference

Household members aged 16 or older whose RDT is valid were considered.

BMI = body mass index; n = number of individuals; OR = odds ratio; CI = confidence interval

*p<0.10 statistical significance

**p<0.05 statistical significance

***p<0.01 statistical significance

#### Multivariate analysis

Logistic regression analysis confirmed that female gender, high BMI, age (being 55 years old or older), and access to a household tap (private or shared) were factors associated with greater seropositivity to SARS-CoV-2 in our study population (see Table 3).

**Table 3:** Factors associated for SARS-CoV-2 infection in the towns of Kombissiri, Manga, and Pô, in the Centre-Sud region of Burkina Faso: Multivariate analysis.

Variables	OR	95% CI	P-value
**Female**	1.48	1.11–1.97.	0.007***
**BMI equal to 25 and above**	1.42	1.04–1.92	0.026**
**55 years old or older**	1.57	1.15–2.14	0.004***
**Access to water** ^**a**^	1.73	1.13–2.67	0.013**
**Constant term (baseline odds)**	0.08	0.06–0.10	0.000***
**N**	2,129		

Household members aged 16 or older whose RDT is valid were considered.

^a^Access to water is an indicator equal to one if the household has access to clean water within dwelling (house, garden or from a neighbor).

BMI = body mass index; n = number of individuals; OR = adjusted Odds ratio by all factors; CI = confidence interval

*p<0.10 statistical significance

**p<0.05 statistical significance

***p<0.01 statistical significance

In addition, we are able to run the same multivariate model varying the dependent variable and using either the probability of carrying IgG antibodies, or that of carrying IgM. IgG reflects anterior infection, while IgM stands for recent or ongoing infections. Results are shown in the S1 and S2 Tables. We found that the covariates remain statistically significant when using the presence of IgG as a dependent variable, while access to water and high BMI lose their significance when using the presence of IgM as a dependent variable. It turns out that in our analysis sample, the IgG prevalence is 9.5% (N = 203/2,143) and the IgM prevalence is 2.6% (N = 55/2,143). Cross tabulation shows that only 5 individuals are positive to IgM and have access to water in their dwelling, and 13 individuals are positive to IgM and have a high BMI. This very small number of cases might drive this non-significance of both covariates. These results are discussed in the next section.

Last, results are unchanged when removing access to water from the set of control variables, as shown in the S3 Table.

## Discussion

The survey revealed a seroprevalence of 11.48% in the population studied, which varied according to town of residence, with the highest seroprevalence observed in Manga (12.86%), followed by Po (11.99%) and Kombissiri (9.35%). This study demonstrates that, despite the limited number of officially reported cases (25 confirmed cases from March 2020 to July 2021, and an additional 4 cases from August to December 2021) [3] the Centre Sud region of Burkina Faso was not spared by the COVID-19 pandemic, with sex, BMI, age, and access to clean water being factors significantly associated with seropositivity. The average seroprevalence observed in the three cities under study is lower than that reported by some researchs conducted in Burkina Faso’s two main cities: between 37.4% and 68.3% in Ouagadougou and between 55.7% and 66.7% in Bobo-Dioulasso [24, 26]. These large cities do not have the same characteristics (population size, spatial occupation, and population socio-cultural and demographic characteristics, standard of living, mobility, trade, population movement, access to preventive and curative healthcare, access to information, financial means to pay for masks, soaps, etc.) as the medium-sized cities covered by our study, so the dynamics of the epidemic are probably different there [15, 30].

Our result was also lower than that reported in Bamako (Mali), in September 2020 [31], where crude seroprevalence rate was 16.5%, and in the Niger State (Nigeria), between June 26 and June 30, 2020 [32], with an average prevalence of 25.41% (21.43% in rural areas). In Africa, most prevalence studies found in our literature review were conducted in the major cities, and mostly targeted specific population groups, particularly those deemed to be at high risk of infection due to their activities or jobs.

In Lomé, a study conducted from April 23 to May 8, 2020, on a group of individuals comprising healthcare workers, police officers, and airport personnel, reported a significantly lower prevalence compared to our study (1.6%; 95% CI: 0.9–2.6%) [33]. Similarly, in Kenya, the prevalence of anti-SARS-CoV-2 IgG antibodies among blood donors tested between April and June 2020 was 5.6% (95% CI: 4.8–6.5%) [34]. A study among blood donors in Quebec suggested that the low prevalence in the study population could be partly explained by self-exclusion bias as people with symptoms may have avoided volunteering to donate blood [35].

**Fig 2:**
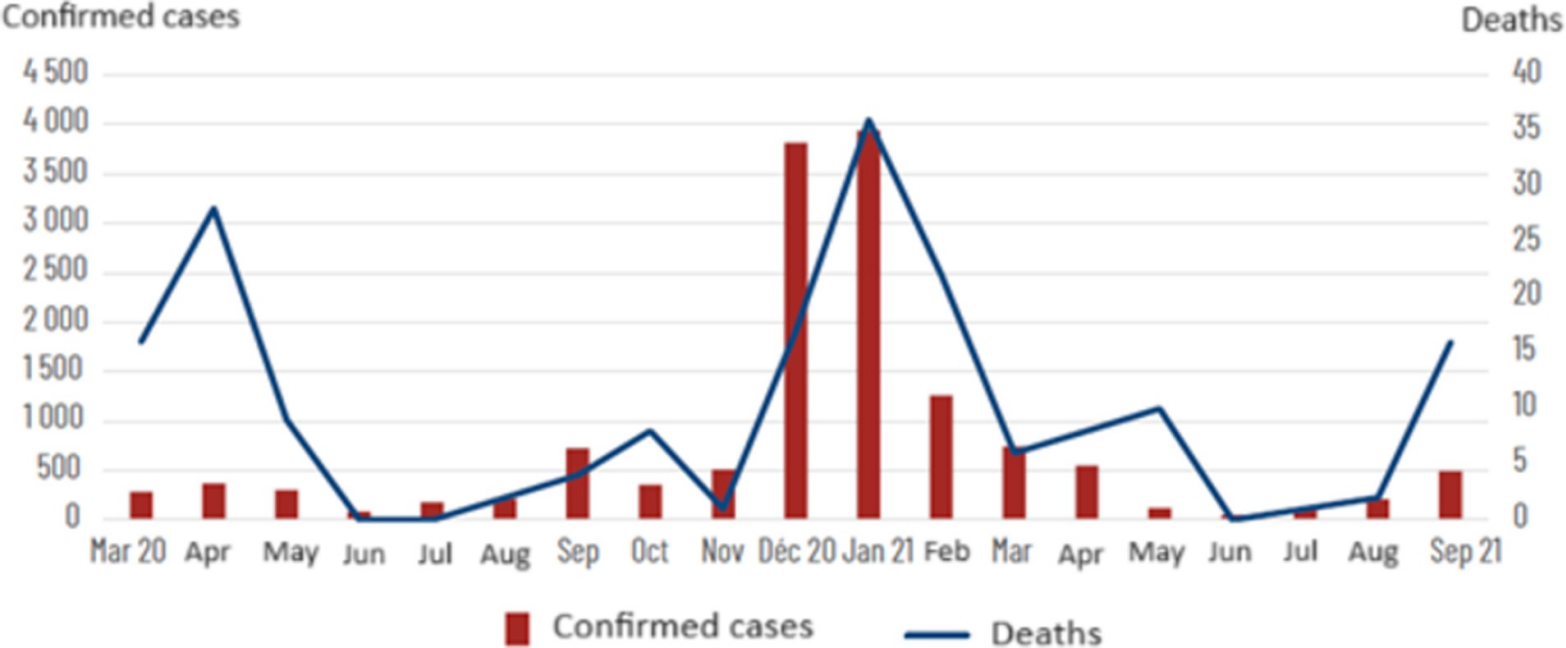
Number of confirmed cases and number of deaths due to COVID-19 infection between March 2020 and September 2021 in Burkina Faso [36].

**Fig 3:**
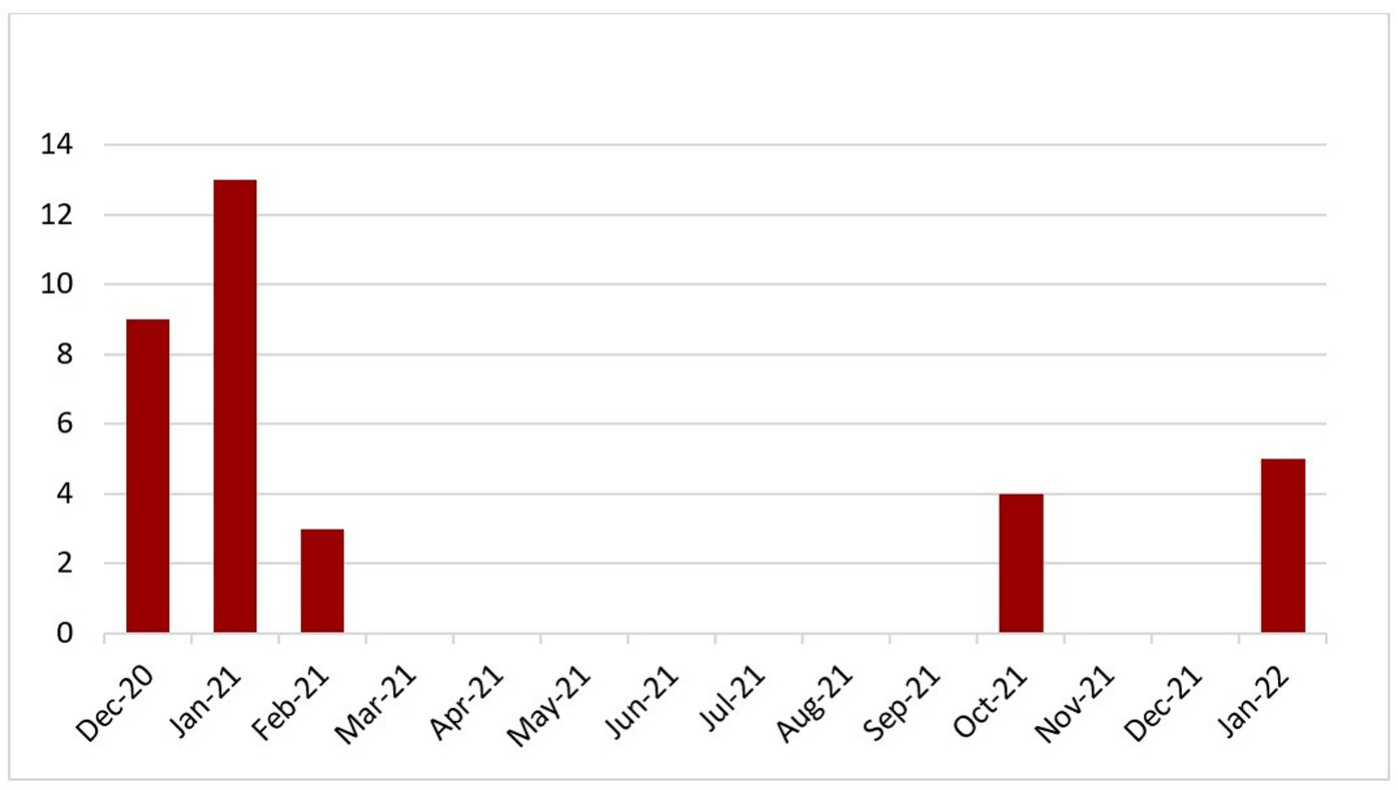
Number of confirmed cases of COVID-19 infection between March 2020 and January 2022 in the region of Centre Sud in Burkina Faso. *Calculations from Burkina Faso Open Data available on https://burkinafaso.opendataforafrica.org/jovpdge/burkina-faso-covid-19-rapport-de-situation [20].

Most of the seroprevalence estimations reported in the literature were conducted during the year 2020 [31, 32–34], while our serological tests were conducted between June and July 2021. Burkina Faso experienced primarily two major waves of infection, the first between September and October 2020, and the second, which was larger, between late November 2020 and February 2021 (see Figs 2 and 3). A timid third wave was observed around June and September 2021.

In addition to variations in the composition of study populations (socio-demographic characteristics), differences in prevalence between studies may be attributed to various factors such as the study period, the study setting (urban or rural), and the type of diagnostic test used (which may vary in sensitivity and specificity). Bobrovitz et al. found that seroprevalence was lower in the general population than in some specific populations, and that national studies had also lower seroprevalence than local studies [37].

In contrast to our study, Halatoko et al. reported a higher risk of SARS-CoV-2 seropositivity among individuals under 55 compared to those aged 55 and over [33], and Bobrovitz found a higher seroprevalence among people aged 18–64 compared to 65 and over [37]. Similar findings were observed in the French SCOPE study, where the differences in the likelihood of infection were attributed to behaviors such as reduced outings and contacts among older adults [38]. Nevertheless, Nwosu et al. found that in Yaounde (Cameroon), seropositivity rates progressively increased with age, ranging from 30.2% among individuals aged 15 to 29 years to 37.5% among subjects over 65 years [39].

These results could be explained by certain physiological predispositions that increase susceptibility to infections in the elderly [39]. In addition, a study conducted in France among participants with an average age of 86 showed that anti-Spike and neutralizing antibodies persisted for at least 9 months after SARS-CoV-2 infection in this age group [40]. Another study [41] reported that the proportion of patients with detectable antibodies titer against SARS-CoV-2 at 180 days was lowest in patients aged 18 to 24 (84.8%; 95% CI: 76.8–93.8%) and highest in those aged 65 and over (96.1%; 95% CI: 92.7–99.8%).

Other researchers also revealed that individuals over 50 or with a body mass index greater than 25 had higher antibody levels compared to others, especially among men, one month after symptom onset [42].

Our study sample excludes people under the age of 16. However, we observe from our survey data the occurrence of symptoms declared on each household member, regardless of their age. Decomposing the sample by age group (below or above 16), we see that the occurrence of any loss of smell over the last three months is lower among individuals below 16 years old compared to others (3.8% vs. 9.2%). The occurrence of any loss of taste is also lower (8.7% vs 17%). This suggests that if these symptoms are related to COVID-19 infection, the seroprevalence rate would have been lower had our protocol not restricted the sample to adults. Furthermore, in Nwosu et al, the test-adjusted seroprevalence estimates for anti-SARS-CoV-2 IgG antibodies is lower among the 5–14 age group (28.9%) than in other age groups (about one percentage point lower than the 15–29 year-olds, and up to 8.6 pp lower that the 65+ year-olds) [39], suggesting also that the average prevalence rate would have been lower if testing had been carried out without age restriction.

Our study indicates higher seroprevalence among females, consistent with findings from the SCOPE and EpiCov studies in France [43]. However, a systematic review of global seroprevalence of SARS-CoV-2 antibodies reported that there was no difference in seroprevalence across sex groups [37]. A study on gender inequalities in COVID-19 conducted in France identified a cross-effect of gender and socio-professional category. Women, due to their employment types, may face higher exposure to SARS-CoV-2 [44]. This prevalence difference across genders could also be attributed to women’s roles in certain communities, particularly in rural areas, where they care for the elderly and the sick, and engage in more frequent outings for visits and errands (e.g., water collection, market visits, income-generating activities) [44]. However, other research indicated that the duration of immunity against SARS-CoV-2 was longer in women than in men after infection [42].

Finally, our study shows a relationship between having access to water within households or compounds and the likelihood of testing positive. We initially expected the opposite relationship, assuming that water access would lead household members to wash their hands more frequently. However, access to water was negatively correlated with poverty risk (p = 0.000) in our data, suggesting that greater access to water is associated with higher socioeconomic status. The observed positive relationship between access to water and prevalence could therefore be explained by the fact that wealthier individuals are more likely to travel to other cities with higher disease prevalence, such as Ouagadougou. Nevertheless, these results should not overshadow the fact that difficulties in accessing clean water have direct health repercussions beyond COVID-19 pandemic, particularly concerning protection against viruses and waterborne diseases. Unfortunately, in some developing countries, water scarcity has been exacerbated due to successive lockdowns, closures, or limited access to public water sources such as fountains and reservoirs. In our sample, only 7% of households had access to piped water at home or from neighbors. Note that the odds ratios of the other risk factors remain unchanged if access to water is excluded from the model (see S1 Table).

### Study strengths

The study benefited from a large sample size and a low refusal rate. The population-based sample included individuals without targeting symptomatic or diagnosed at-risk populations.

### Study limitations

The main limitations of the study were the period of data collection (too late after the second wave to detect people infected during that peak) and the diagnostic performance of the rapid test used in an African population. One concern of the study is the lack of a clear understanding of the Biosynex COVID-19 BSS and other rapid tests’ diagnostic performance. The performance of the test mentioned above is calculated on samples collected from French patients, hence diagnostic performances might differ in African populations, in particular due to population-specific cross-reactivity. A study by Ouedraogo et al. estimated the specificity of the Biosynex COVID-19 BSS rapid test at 99.36% and sensitivity at 48.41%, compared to the WANTAI SARS CoV-2 Ab ELISA immunoassay as the reference test, suggesting an underestimation of seroprevalence in our study population [45]. Yet, it has also been shown that ELISA immunoassays can have relatively low specificity in West African populations [46, 47]. This raises concerns that estimating the sensitivity of rapid antibody tests from comparisons with ELISA immunoassays might underestimate the relative sensitivity of these tests.

Indeed, a validation study conducted in South Africa with RT-PCR as the reference standard reports a sensitivity of 84.3% for the Biosynex COVID-19 BSS and does not confirm the systematically low diagnostic accuracy reported by Ouedraogo et al. for rapid serological tests in general [48]. Finally, the Hangzhou Biotest Biotech CoronaCHEK [49] is another immunochromatographic test which has the same target as the Biosynex BSS, the receptor-binding domain of the spike surface protein of SARS-CoV-2, and has been shown to have sensitivity above 80% when using RT-PCR confirmed specimens in African populations. The extent of the underestimation of seroprevalence rates in our study therefore remains unclear in the absence of a validation of rapid antibody tests, and in particular of the Biosynex BSS, with RT-PCR as the reference method in Burkina Faso, our study population.

## Conclusions

The results of this study indicate that COVID-19 has been circulating more extensively within middle-sized cities in Burkina Faso than that reported by the health authorities, given the low use of systematic screening. Prevention measures implemented to contain the pandemic must take these areas into consideration. Our findings highlight the vulnerability of women, older adults, and overweight and obese individuals to the epidemic.

## Supporting information

S1 Table Factors associated for SARS-CoV-2 infection (presence of IgG) in the towns of Kombissiri, Manga, and Pô, in the Centre-Sud region of Burkina Faso: Multivariate analysis. (DOCX)

S2 Table Factors associated for SARS-CoV-2 infection (presence of IgM) in the towns of Kombissiri, Manga, and Pô, in the Centre-Sud region of Burkina Faso: Multivariate analysis. (DOCX)

S3 Table Factors associated for SARS-CoV-2 infection in the towns of Kombissiri, Manga, and Pô, in the Centre-Sud region of Burkina Faso: Multivariate analysis (not controlling for access to water). (DOCX)
