# Supplementary material for: Seroprevalence and risk factors for SARS-CoV-2 infection in middle-sized cities of Burkina Faso: A descriptive cross-sectional study
Source: PLoS One. 2024 Aug 7;19(8):e0305850. doi: 10.1371/journal.pone.0305850 (PMC11305540; doi:10.1371/journal.pone.0305850)
Supplement: S2 Table — (DOCX) [file pone.0305850.s002.docx]

**S2 Table: Factors associated for SARS-CoV-2 infection (presence of IgM) in the towns of Kombissiri, Manga, and Pô, in the Centre-Sud region of Burkina Faso : multivariate analysis**

| **Variables** | **OR** | **95% CI** | **P-value** |
| --- | --- | --- | --- |
| **Female** | 3.47 | 1.73 - 6.97 | 0.000*** |
| **BMI equal to 25 and above** | 0.93 | 0.49 - 1.76 | 0.814 |
| **55 years old or older** | 2.00 | 1.12 - 3.58 | 0.019** |
| **Access to water** | 1.23 | 0.48 - 3.17 | 0.666 |
| **Constant term (baseline odds)** | 0.01 | 0.004 - 0.02 | 0.000*** |
| **n** | 2,129 |  |  |

*Notes*. Sample : Household members aged 16 or older whose RDT is valid. BMI body mass index, n number of individuals, OR adjusted Odds ratio by all factors, CI confidence interval, RDT rapid diagnostic test.

*p<0.10 statistical significance

**p<0.05 statistical significance

***p<0.01 statistical significance
