# Supplementary material for: Seroprevalence and risk factors for SARS-CoV-2 infection in middle-sized cities of Burkina Faso: A descriptive cross-sectional study
Source: PLoS One. 2024 Aug 7;19(8):e0305850. doi: 10.1371/journal.pone.0305850 (PMC11305540; doi:10.1371/journal.pone.0305850)
Supplement: S3 Table — (DOCX) [file pone.0305850.s003.docx]

**S3 Table: Factors associated for SARS-CoV-2 infection in the towns of Kombissiri, Manga, and Pô, in the Centre-Sud region of Burkina Faso : multivariate analysis (not controlling for access to water)**

| **Variables** | **OR** | **95% CI** | **P-value** |
| --- | --- | --- | --- |
| **Female** | 1.47 | 1.11-1.96 | 0.008*** |
| **BMI equal to 25 and above** | 1.46 | 1.07-1.98 | 0.015** |
| **55 years old or older** | 1.60 | 1.17-2.18 | 0.003*** |
| **Constant term (baseline odds)** | 0.08 | 0.07-0.11 | 0.000*** |
| **n** | 2,141 |  |  |

*Notes*. Sample : Household members aged 16 or older whose RDT is valid. BMI body mass index, n number of individuals, OR adjusted Odds ratio by all factors, CI confidence interval, RDT rapid diagnostic test.

*p<0.10 statistical significance

**p<0.05 statistical significance

***p<0.01 statistical significance
